# Crystal structure of (*E*)-2-({[2-(1,3-dioxan-2-yl)phen­yl]imino}­meth­yl)phenol

**DOI:** 10.1107/S2056989015008051

**Published:** 2015-04-30

**Authors:** Zhengyi Li, Song Shi, Kun Zhou, Liang Chen, Xiaoqiang Sun

**Affiliations:** a Jiangsu Key Laboratory of Advanced Catalytic Materials and Technology, School of Petrochemical Engineering, Changzhou University, Changzhou 213164, Jiangsu, People’s Republic of China

**Keywords:** crystal structure, acetal, Schiff base, intra­molecular hydrogen bonding, N—H⋯O hydrogen bonds

## Abstract

The title compound, C_17_H_17_NO_3_, prepared by the condensation reaction of 2-(1,3-dioxan-2-yl)aniline and salicyl­aldehyde, has an *E* conformation about the C=N bond. The six-membered O-heterocycle adopts a chair conformation, with the bond to the aromatic ring located at its equatorial position. The dihedral angle between the aromatic rings is 36.54 (9)°. There is an intra­molecular N—H⋯O hydrogen bond forming an *S*(6) ring motif. In the crystal, mol­ecules are linked by C—H⋯O hydrogen bonds, forming chains along the *a*-axis direction. Within the chains, there are C—H⋯π inter­actions involving adjacent mol­ecules.

## Related literature   

For general background to acetals, see: Cismaş *et al.* (2005 ▸); Sun *et al.* (2010 ▸). For Schiff bases of salicyl­aldehyde having important applications in biological and pharmacological chemistry, see: Gupta & Sutar (2008 ▸); Jiménez-Sánchez *et al.* (2013 ▸). For further background to related Schiff base ligands and their various properties, see: Arod *et al.* (2005 ▸); Chatziefthimiou *et al.* (2006 ▸).
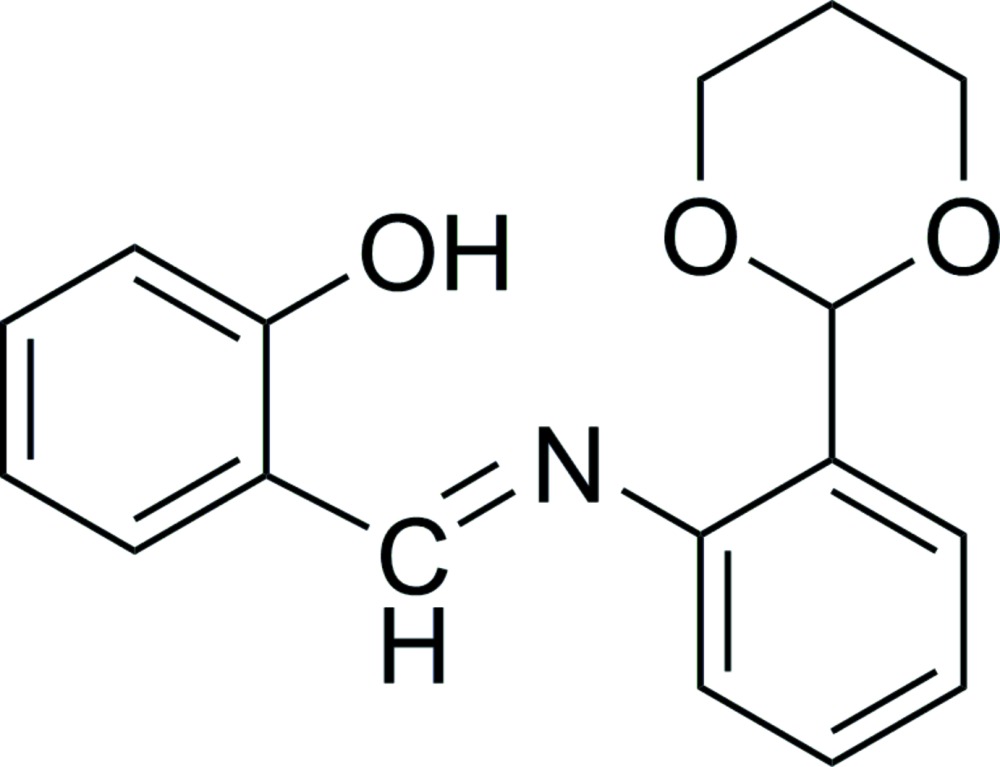


## Experimental   

### Crystal data  

C_17_H_17_NO_3_
*M* *_r_* = 283.32
Orthorhombic, 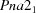

*a* = 8.4873 (18) Å
*b* = 10.821 (2) Å
*c* = 16.232 (3) Å
*V* = 1490.8 (5) Å^3^
*Z* = 4
Mo *K*α radiation
μ = 0.09 mm^−1^
*T* = 296 K
0.26 × 0.24 × 0.22 mm

### Data collection  

Bruker APEXII CCD area-detector diffractometer
Absorption correction: multi-scan (*SADABS*; Bruker, 2009 ▸) *T*_min_ = 0.978, *T*_max_ = 0.981
9005 measured reflections
3123 independent reflections
2494 reflections with *I* > 2σ(*I*)
*R*_int_ = 0.043

### Refinement  

*R*[*F* ^2^ > 2σ(*F* ^2^)] = 0.039
*wR*(*F* ^2^) = 0.097
*S* = 1.00
3123 reflections
190 parameters
1 restraint
H-atom parameters constrained
Δρ_max_ = 0.15 e Å^−3^
Δρ_min_ = −0.21 e Å^−3^

### 

Data collection: *APEX2* (Bruker, 2009 ▸); cell refinement: *SAINT* (Bruker, 2009 ▸); data reduction: *SAINT*; program(s) used to solve structure: *SHELXS97* (Sheldrick, 2008 ▸); program(s) used to refine structure: *SHELXL97* (Sheldrick, 2008 ▸); molecular graphics: *SHELXTL* (Sheldrick, 2008 ▸); software used to prepare material for publication: *SHELXTL*.

## Supplementary Material

Crystal structure: contains datablock(s) I, Global. DOI: 10.1107/S2056989015008051/su5122sup1.cif

Structure factors: contains datablock(s) I. DOI: 10.1107/S2056989015008051/su5122Isup2.hkl

Supporting information file. DOI: 10.1107/S2056989015008051/su5122Isup3.cml

**Fig. 1.**
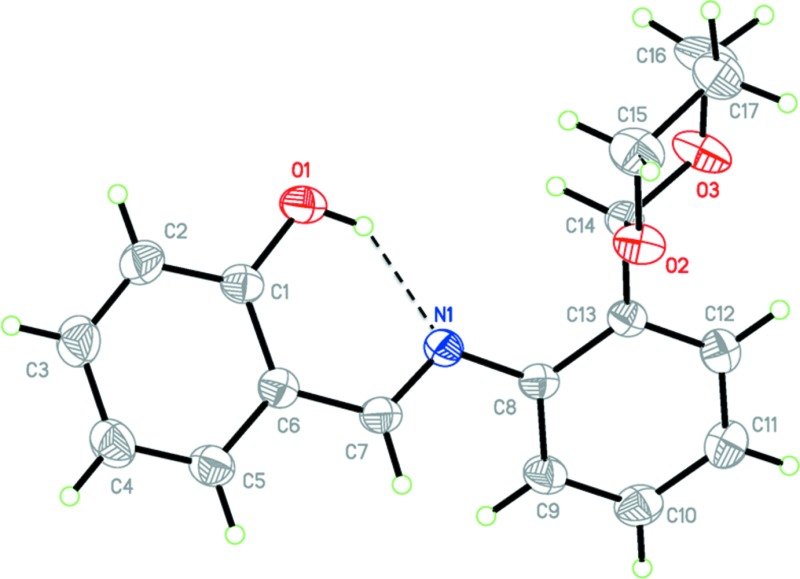
The molecular structure of the title compound, with atom labelling. Displacement ellipsoids are drawn at the 30% probability level. The intramolecular O—H···N hydrogen bond is shown as a dashed line (see Table 1 for details).

**Fig. 2.**
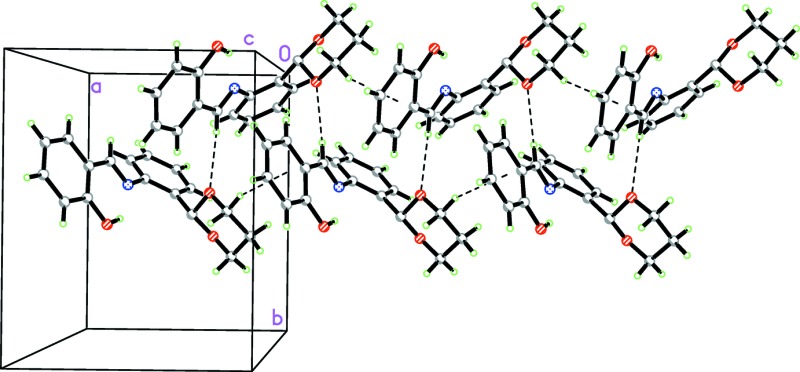
A partial view of the crystal packing of the title compound, view along the c axis. Hydrogen bonds are shown as dashed lines (see Table 1 for details).

. DOI: 10.1107/S2056989015008051/su5122fig1.tif
The mol­ecular structure of the title compound, with atom labelling. Displacement ellipsoids are drawn at the 30% probability level. The intra­molecular O—H⋯N hydrogen bond is shown as a dashed line (see Table 1 for details).

c . DOI: 10.1107/S2056989015008051/su5122fig2.tif
A partial view of the crystal packing of the title compound, view along the *c* axis. Hydrogen bonds are shown as dashed lines (see Table 1 for details).

CCDC reference: 1061272

Additional supporting information:  crystallographic information; 3D view; checkCIF report

## Figures and Tables

**Table 1:** Hydrogen-bond geometry (, ) *Cg*1 is the centroid of the C1C6 ring.

*D*H*A*	*D*H	H*A*	*D* *A*	*D*H*A*
O1H1N1	0.85	1.90	2.632(2)	144
C7H7O2^i^	0.93	2.48	3.364(2)	160
C15H15*A* *Cg*1^ii^	0.97	2.77	3.694(3)	160

Symmetry codes: (i) 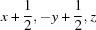; (ii) 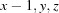.

## supplementary crystallographic information

### S1. Comment

Schiff bases of salicyl­aldehyde have important applications in biological chemistry and pharmacological chemistry (Gupta *et al.*, 2008; Sánchez *et al.*, 2013). In addition, Schiff bases of salicyl­aldehyde has good optical properties with the ability of distinctive ultraviolet absorption (Chatziefthimiou *et al.*, 2006). Herein (E)-2-{[2-(1,3-dioxan-2-yl) phenyl) imino]­methyl} phenol was prepared by the condensation reaction of 2-(1,3-dioxan-2-yl) aniline and salicyl­aldehyde, and the structure was confirmed by X-ray diffraction analysis.

In the molecular structure of the title compound, Fig. 1, the two aromatic rings (C1—C6 and C8—C13) are linked by the double bond C7═N1, with the dihedral angle between the two rings being 36.54 (9) °. The C7═N1 bond is coplanar with the benzene ring (C1—C6), and atom N1 forms an intra­molecular hydrogen bond, O1—H1···N1, with the hydroxyl group on ring (C1—C6) [Fig. 1 and Table 1]. The six-membered O-heterocycle adopts a chair conformation with the (C8—C13) ring located at its equatorial position.

In the crystal, molecules are linked by C—H···O hydrogen bonds forming chain along the *a* axis. Within the chains there are C—H···π inter­actions involving adjacent molecules (Table 1 and Fig. 2).

### S2. Experimental

A mixture of 2-(1,3-dioxan-2-yl) aniline (1.8 g, 10 mmol) and salicyl­aldehyde (1.32 g, 11 mmol) in 20 ml methanol, stirred for 20 h at room temperature. After the reaction had finished, the solution was left overnight at at 273 K, and yellow block-like crystals were obtained on slow evaporation of the solvent (yield: 82%; m.p.: 321 K).

### S3. Refinement

The OH and C-bound H atoms were placed in geometrically idealized positions and constrained to ride on their parent atoms: O—H = 0.85 Å, C—H = 0.93-0.98 Å, with *U*_iso_(H) = 1.5*U*_eq_(O) and 1.2U_eq_(C) for other H atoms.

### Crystal data

**Table:**

C_17_H_17_NO_3_	*F*(000) = 600
*M**_r_* = 283.32	*D*_x_ = 1.262 Mg m^−^^3^
Orthorhombic, *P**n**a*2_1_	Mo *K*α radiation, λ = 0.71073 Å
Hall symbol: P 2c -2n	Cell parameters from 3792 reflections
*a* = 8.4873 (18) Å	θ = 2.3–26.4°
*b* = 10.821 (2) Å	µ = 0.09 mm^−^^1^
*c* = 16.232 (3) Å	*T* = 296 K
*V* = 1490.8 (5) Å^3^	Block, yellow
*Z* = 4	0.26 × 0.24 × 0.22 mm

### Data collection

**Table:**

Bruker APEXII CCD area-detector diffractometer	3123 independent reflections
Radiation source: fine-focus sealed tube	2494 reflections with *I* > 2σ(*I*)
Graphite monochromator	*R*_int_ = 0.043
phi and ω scans	θ_max_ = 27.7°, θ_min_ = 2.3°
Absorption correction: multi-scan (*SADABS*; Bruker, 2009)	*h* = −10→11
*T*_min_ = 0.978, *T*_max_ = 0.981	*k* = −13→13
9005 measured reflections	*l* = −17→21

### Refinement

**Table:**

Refinement on *F*^2^	Primary atom site location: structure-invariant direct methods
Least-squares matrix: full	Secondary atom site location: difference Fourier map
*R*[*F*^2^ > 2σ(*F*^2^)] = 0.039	Hydrogen site location: inferred from neighbouring sites
*wR*(*F*^2^) = 0.097	H-atom parameters constrained
*S* = 1.00	*w* = 1/[σ^2^(*F*_o_^2^) + (0.0565*P*)^2^ + 0.020*P*] where *P* = (*F*_o_^2^ + 2*F*_c_^2^)/3
3123 reflections	(Δ/σ)_max_ < 0.001
190 parameters	Δρ_max_ = 0.15 e Å^−^^3^
1 restraint	Δρ_min_ = −0.21 e Å^−^^3^

### Special details

**Table:**

Geometry. All e.s.d.'s (except the e.s.d. in the dihedral angle between two l.s. planes) are estimated using the full covariance matrix. The cell e.s.d.'s are taken into account individually in the estimation of e.s.d.'s in distances, angles and torsion angles; correlations between e.s.d.'s in cell parameters are only used when they are defined by crystal symmetry. An approximate (isotropic) treatment of cell e.s.d.'s is used for estimating e.s.d.'s involving l.s. planes.
Refinement. Refinement of *F*^2^ against ALL reflections. The weighted *R*-factor *wR* and goodness of fit *S* are based on *F*^2^, conventional *R*-factors *R* are based on *F*, with *F* set to zero for negative *F*^2^. The threshold expression of *F*^2^ > σ(*F*^2^) is used only for calculating *R*-factors(gt) *etc*. and is not relevant to the choice of reflections for refinement. *R*-factors based on *F*^2^ are statistically about twice as large as those based on *F*, and *R*- factors based on ALL data will be even larger.

### Fractional atomic coordinates and isotropic or equivalent isotropic displacement parameters (Å^2^)

**Table:**

	*x*	*y*	*z*	*U*_iso_*/*U*_eq_	
C1	0.36289 (19)	0.02306 (15)	0.41243 (12)	0.0456 (4)	
C2	0.4402 (2)	0.0087 (2)	0.48668 (13)	0.0594 (5)	
H2	0.4360	−0.0668	0.5140	0.071*	
C3	0.5234 (3)	0.1054 (2)	0.52064 (14)	0.0666 (6)	
H3	0.5749	0.0945	0.5707	0.080*	
C4	0.5314 (2)	0.2185 (2)	0.48125 (14)	0.0645 (5)	
H4	0.5872	0.2835	0.5047	0.077*	
C5	0.4561 (2)	0.23357 (17)	0.40717 (14)	0.0567 (5)	
H5	0.4616	0.3096	0.3807	0.068*	
C6	0.37108 (19)	0.13744 (15)	0.37051 (12)	0.0442 (4)	
C7	0.2987 (2)	0.15509 (15)	0.29062 (12)	0.0455 (4)	
H7	0.3059	0.2324	0.2659	0.055*	
C8	0.16825 (19)	0.09047 (14)	0.17144 (11)	0.0409 (4)	
C9	0.2536 (2)	0.16144 (17)	0.11485 (13)	0.0507 (4)	
H9	0.3494	0.1962	0.1303	0.061*	
C10	0.1966 (2)	0.18018 (19)	0.03623 (13)	0.0572 (5)	
H10	0.2533	0.2288	−0.0005	0.069*	
C11	0.0567 (2)	0.12752 (18)	0.01177 (12)	0.0549 (5)	
H11	0.0185	0.1407	−0.0412	0.066*	
C12	−0.0266 (2)	0.05486 (16)	0.06659 (12)	0.0482 (4)	
H12	−0.1208	0.0188	0.0499	0.058*	
C13	0.02793 (18)	0.03466 (13)	0.14640 (10)	0.0390 (3)	
C14	−0.06859 (19)	−0.03996 (13)	0.20547 (11)	0.0415 (4)	
H14	0.0019	−0.0855	0.2424	0.050*	
C15	−0.2564 (3)	−0.0245 (2)	0.31050 (16)	0.0704 (6)	
H15A	−0.3223	0.0334	0.3404	0.084*	
H15B	−0.1891	−0.0664	0.3499	0.084*	
C16	−0.3585 (2)	−0.1176 (2)	0.26691 (17)	0.0710 (6)	
H16A	−0.4128	−0.1685	0.3071	0.085*	
H16B	−0.4371	−0.0752	0.2340	0.085*	
C17	−0.2593 (3)	−0.1966 (2)	0.21304 (19)	0.0797 (7)	
H17A	−0.1945	−0.2500	0.2470	0.096*	
H17B	−0.3267	−0.2486	0.1794	0.096*	
N1	0.22509 (16)	0.06900 (12)	0.25250 (9)	0.0427 (3)	
O1	0.28135 (16)	−0.07360 (11)	0.38050 (9)	0.0606 (4)	
H1	0.2368	−0.0545	0.3354	0.091*	
O2	−0.16132 (15)	0.04108 (10)	0.25196 (9)	0.0536 (3)	
O3	−0.16055 (18)	−0.12428 (12)	0.16085 (10)	0.0670 (4)	

### Atomic displacement parameters (Å^2^)

**Table:**

	*U*^11^	*U*^22^	*U*^33^	*U*^12^	*U*^13^	*U*^23^
C1	0.0412 (9)	0.0476 (8)	0.0479 (9)	−0.0025 (7)	0.0094 (8)	0.0012 (7)
C2	0.0610 (11)	0.0654 (11)	0.0517 (11)	−0.0032 (10)	0.0004 (9)	0.0117 (10)
C3	0.0688 (13)	0.0826 (14)	0.0484 (11)	−0.0044 (11)	−0.0071 (10)	0.0028 (11)
C4	0.0687 (12)	0.0662 (11)	0.0586 (13)	−0.0109 (10)	−0.0074 (10)	−0.0122 (10)
C5	0.0586 (11)	0.0474 (9)	0.0642 (12)	−0.0064 (8)	−0.0016 (10)	−0.0024 (9)
C6	0.0400 (8)	0.0445 (8)	0.0480 (9)	0.0015 (7)	0.0043 (8)	−0.0020 (7)
C7	0.0459 (9)	0.0382 (7)	0.0525 (10)	−0.0014 (7)	0.0030 (8)	0.0037 (7)
C8	0.0410 (8)	0.0364 (7)	0.0452 (9)	−0.0001 (6)	0.0038 (7)	0.0011 (7)
C9	0.0460 (9)	0.0530 (9)	0.0531 (11)	−0.0096 (8)	0.0056 (8)	0.0053 (8)
C10	0.0583 (11)	0.0591 (10)	0.0542 (11)	−0.0029 (9)	0.0128 (9)	0.0105 (8)
C11	0.0559 (11)	0.0643 (11)	0.0446 (10)	0.0081 (9)	0.0046 (8)	0.0013 (9)
C12	0.0428 (9)	0.0521 (9)	0.0496 (10)	0.0037 (7)	0.0009 (8)	−0.0063 (8)
C13	0.0376 (8)	0.0335 (7)	0.0460 (9)	0.0031 (6)	0.0042 (7)	−0.0044 (6)
C14	0.0361 (8)	0.0364 (7)	0.0519 (9)	0.0016 (6)	0.0014 (7)	0.0004 (7)
C15	0.0669 (13)	0.0810 (14)	0.0632 (14)	−0.0131 (12)	0.0203 (11)	−0.0020 (12)
C16	0.0464 (10)	0.0842 (13)	0.0824 (16)	−0.0132 (10)	0.0135 (11)	0.0118 (13)
C17	0.0778 (15)	0.0588 (11)	0.103 (2)	−0.0284 (11)	0.0238 (14)	−0.0027 (13)
N1	0.0381 (7)	0.0429 (6)	0.0470 (8)	−0.0035 (5)	0.0011 (7)	0.0035 (6)
O1	0.0697 (8)	0.0510 (6)	0.0612 (8)	−0.0186 (6)	−0.0049 (7)	0.0099 (7)
O2	0.0570 (7)	0.0468 (6)	0.0570 (7)	−0.0036 (5)	0.0189 (6)	−0.0069 (6)
O3	0.0723 (9)	0.0561 (7)	0.0726 (10)	−0.0275 (7)	0.0215 (8)	−0.0191 (7)

### Geometric parameters (Å, º)

**Table:**

C1—O1	1.357 (2)	C10—H10	0.9300
C1—C2	1.381 (3)	C11—C12	1.382 (3)
C1—C6	1.414 (2)	C11—H11	0.9300
C2—C3	1.378 (3)	C12—C13	1.393 (3)
C2—H2	0.9300	C12—H12	0.9300
C3—C4	1.382 (3)	C13—C14	1.497 (2)
C3—H3	0.9300	C14—O2	1.399 (2)
C4—C5	1.372 (3)	C14—O3	1.402 (2)
C4—H4	0.9300	C14—H14	0.9800
C5—C6	1.399 (3)	C15—O2	1.435 (2)
C5—H5	0.9300	C15—C16	1.505 (3)
C6—C7	1.448 (3)	C15—H15A	0.9700
C7—N1	1.281 (2)	C15—H15B	0.9700
C7—H7	0.9300	C16—C17	1.485 (3)
C8—C13	1.396 (2)	C16—H16A	0.9700
C8—C9	1.399 (2)	C16—H16B	0.9700
C8—N1	1.420 (2)	C17—O3	1.426 (3)
C9—C10	1.380 (3)	C17—H17A	0.9700
C9—H9	0.9300	C17—H17B	0.9700
C10—C11	1.375 (3)	O1—H1	0.8501
			
O1—C1—C2	119.27 (16)	C11—C12—H12	119.4
O1—C1—C6	121.05 (17)	C13—C12—H12	119.4
C2—C1—C6	119.67 (17)	C12—C13—C8	119.10 (15)
C3—C2—C1	120.50 (19)	C12—C13—C14	119.90 (14)
C3—C2—H2	119.8	C8—C13—C14	120.91 (15)
C1—C2—H2	119.8	O2—C14—O3	111.92 (14)
C2—C3—C4	120.8 (2)	O2—C14—C13	108.36 (11)
C2—C3—H3	119.6	O3—C14—C13	108.94 (15)
C4—C3—H3	119.6	O2—C14—H14	109.2
C5—C4—C3	119.21 (19)	O3—C14—H14	109.2
C5—C4—H4	120.4	C13—C14—H14	109.2
C3—C4—H4	120.4	O2—C15—C16	110.1 (2)
C4—C5—C6	121.66 (19)	O2—C15—H15A	109.6
C4—C5—H5	119.2	C16—C15—H15A	109.6
C6—C5—H5	119.2	O2—C15—H15B	109.6
C5—C6—C1	118.13 (17)	C16—C15—H15B	109.6
C5—C6—C7	120.13 (16)	H15A—C15—H15B	108.2
C1—C6—C7	121.71 (15)	C17—C16—C15	109.61 (17)
N1—C7—C6	122.95 (15)	C17—C16—H16A	109.7
N1—C7—H7	118.5	C15—C16—H16A	109.7
C6—C7—H7	118.5	C17—C16—H16B	109.7
C13—C8—C9	119.22 (16)	C15—C16—H16B	109.7
C13—C8—N1	119.25 (14)	H16A—C16—H16B	108.2
C9—C8—N1	121.46 (15)	O3—C17—C16	111.56 (16)
C10—C9—C8	120.40 (18)	O3—C17—H17A	109.3
C10—C9—H9	119.8	C16—C17—H17A	109.3
C8—C9—H9	119.8	O3—C17—H17B	109.3
C11—C10—C9	120.59 (18)	C16—C17—H17B	109.3
C11—C10—H10	119.7	H17A—C17—H17B	108.0
C9—C10—H10	119.7	C7—N1—C8	119.61 (14)
C10—C11—C12	119.44 (19)	C1—O1—H1	111.6
C10—C11—H11	120.3	C14—O2—C15	111.33 (13)
C12—C11—H11	120.3	C14—O3—C17	112.17 (17)
C11—C12—C13	121.21 (17)		
			
O1—C1—C2—C3	−179.79 (19)	C9—C8—C13—C12	−2.4 (2)
C6—C1—C2—C3	0.8 (3)	N1—C8—C13—C12	−179.32 (15)
C1—C2—C3—C4	0.0 (3)	C9—C8—C13—C14	−178.78 (15)
C2—C3—C4—C5	−0.4 (3)	N1—C8—C13—C14	4.3 (2)
C3—C4—C5—C6	0.1 (3)	C12—C13—C14—O2	−94.14 (18)
C4—C5—C6—C1	0.7 (3)	C8—C13—C14—O2	82.23 (17)
C4—C5—C6—C7	−177.36 (18)	C12—C13—C14—O3	27.85 (19)
O1—C1—C6—C5	179.50 (15)	C8—C13—C14—O3	−155.78 (15)
C2—C1—C6—C5	−1.1 (2)	O2—C15—C16—C17	52.4 (3)
O1—C1—C6—C7	−2.5 (3)	C15—C16—C17—O3	−51.1 (3)
C2—C1—C6—C7	176.88 (16)	C6—C7—N1—C8	−174.91 (15)
C5—C6—C7—N1	177.01 (16)	C13—C8—N1—C7	−146.14 (15)
C1—C6—C7—N1	−1.0 (3)	C9—C8—N1—C7	37.0 (2)
C13—C8—C9—C10	2.6 (3)	O3—C14—O2—C15	60.3 (2)
N1—C8—C9—C10	179.47 (18)	C13—C14—O2—C15	−179.59 (16)
C8—C9—C10—C11	−1.3 (3)	C16—C15—O2—C14	−57.2 (2)
C9—C10—C11—C12	−0.3 (3)	O2—C14—O3—C17	−58.6 (2)
C10—C11—C12—C13	0.5 (3)	C13—C14—O3—C17	−178.37 (16)
C11—C12—C13—C8	0.9 (2)	C16—C17—O3—C14	54.4 (3)
C11—C12—C13—C14	177.32 (15)		

### Hydrogen-bond geometry (Å, º)

Cg1 is the centroid of the C1–C6 ring.

**Table:**

*D*—H···*A*	*D*—H	H···*A*	*D*···*A*	*D*—H···*A*
O1—H1···N1	0.85	1.90	2.632 (2)	144
C7—H7···O2^i^	0.93	2.48	3.364 (2)	160
C15—H15*A*···*Cg*1^ii^	0.97	2.77	3.694 (3)	160

Symmetry codes: (i) *x*+1/2, −*y*+1/2, *z*; (ii) *x*−1, *y*, *z*.
