# Supplementary figures and images for: Crystal structure of (E)-2-({[2-(1,3-dioxan-2-yl)phen­yl]imino}­meth­yl)phenol
Source: Acta Crystallogr E Crystallogr Commun. 2015 Apr 30;71(Pt 5):o357–8. doi: 10.1107/S2056989015008051 (PMC4420126; doi:10.1107/S2056989015008051)

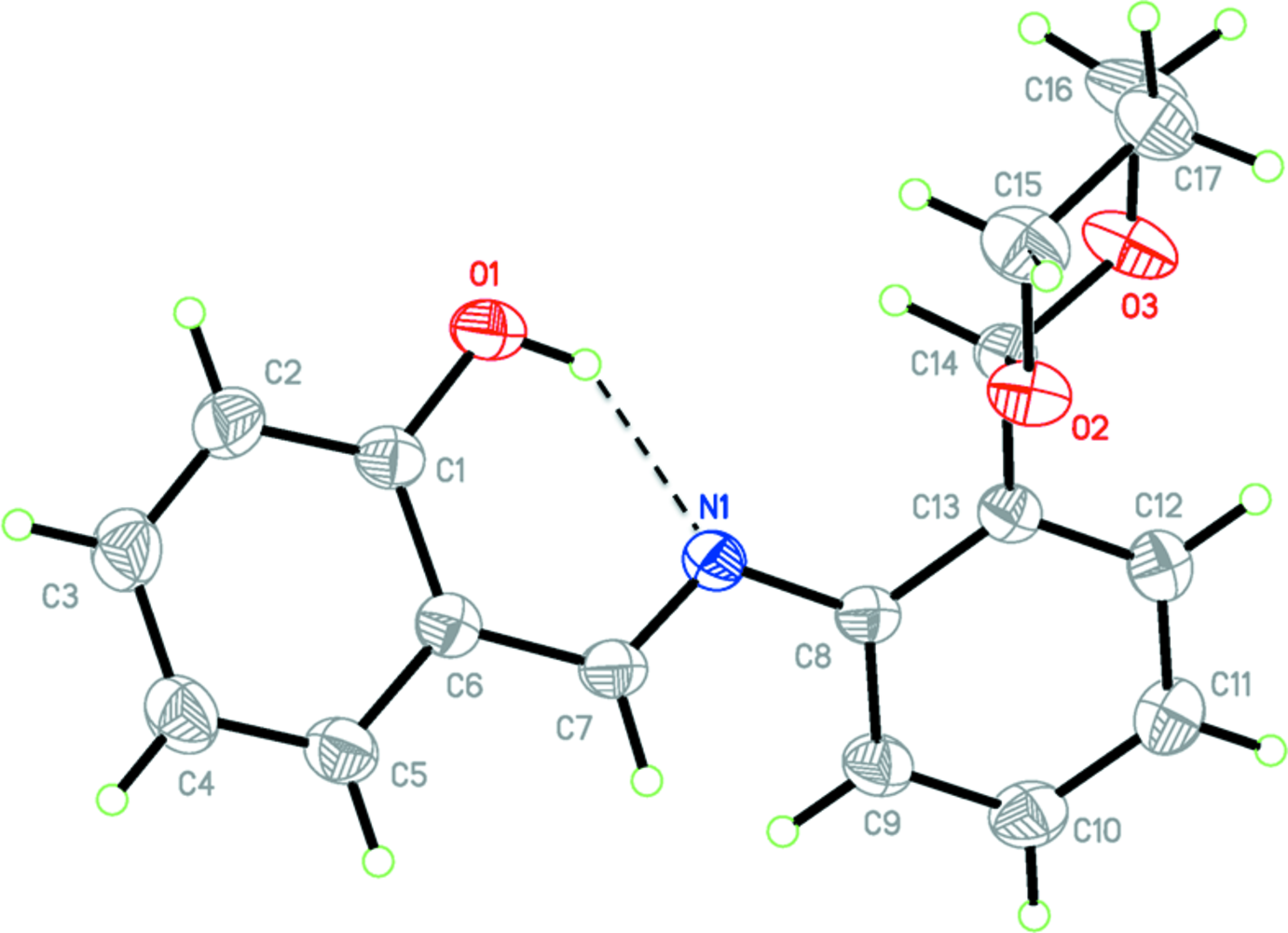

Supplement: Supplementary file 4 [file e-71-0o357-fig1.tif]

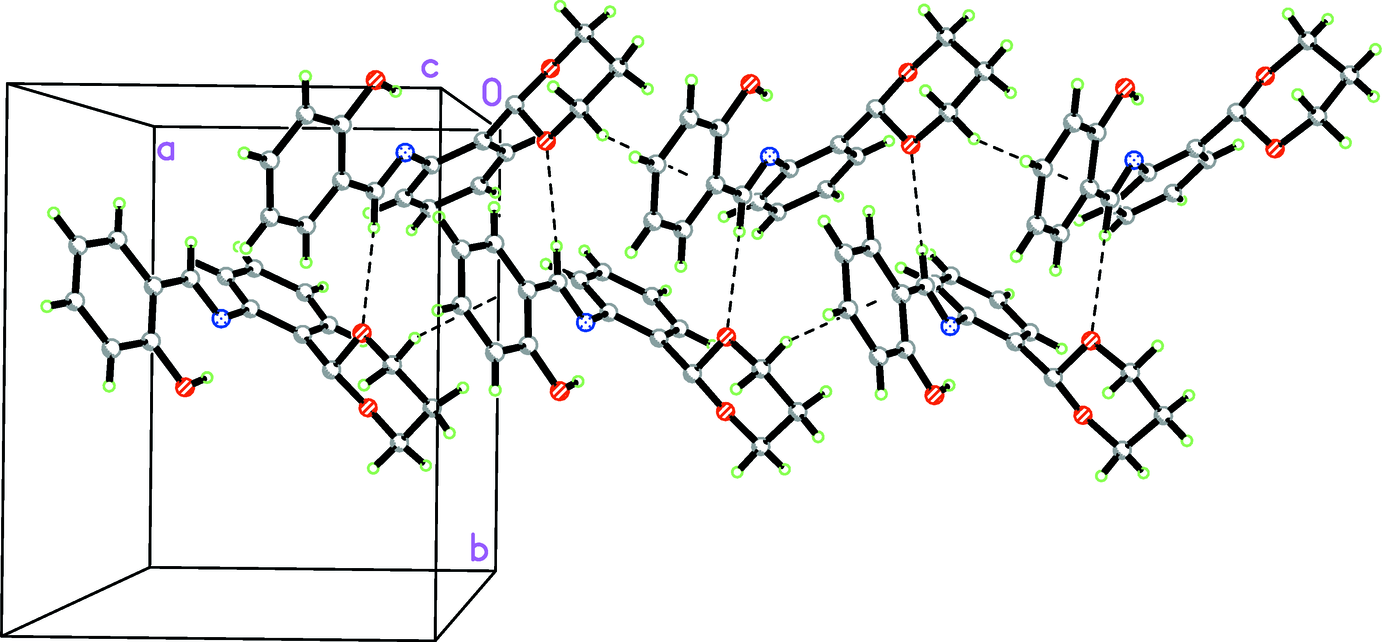

Supplement: Supplementary file 5 [file e-71-0o357-fig2.tif]
